# Global Annotation, Expression Analysis, and Stability of Candidate sRNAs in Group B Streptococcus

**DOI:** 10.1128/mBio.02803-21

**Published:** 2021-11-02

**Authors:** Rebecca A. Keogh, Brady L. Spencer, Hailee M. Sorensen, Rachel L. Zapf, Paul Briaud, Abigail E. Bonsall, Kelly S. Doran, Ronan K. Carroll

**Affiliations:** a Department of Immunology and Microbiology, University of Colorado Anschutz, Aurora, Colorado, USA; b Department of Biological Sciences, Ohio University, Athens, Ohio, USA; University of Illinois at Chicago

**Keywords:** RNA stability, *Streptococcus agalactiae*, group B streptococcus, regulatory RNA, sRNA, virulence

## Abstract

Small, noncoding RNAs (sRNAs) are being increasingly identified as important regulatory molecules in prokaryotes. Due to the prevalence of next-generation sequencing-based techniques, such as RNA sequencing (RNA-seq), there is potential for increased discovery of sRNAs within bacterial genomes; however, these elements are rarely included in annotation files. Consequently, expression values for sRNAs are omitted from most transcriptomic analyses, and mechanistic studies have lagged behind those of protein regulators in numerous bacteria. Two previous studies have identified sRNAs in the human pathogen group B Streptococcus (GBS). Here, we utilize the data from these studies to create updated genome annotation files for the model GBS strains NEM316 and COH1. Using the updated COH1 annotation file, we reanalyze publicly available GBS RNA-seq whole-transcriptome data from GenBank to monitor GBS sRNA expression under a variety of conditions and genetic backgrounds. This analysis generated expression values for 232 putative sRNAs that were overlooked in previous transcriptomic analyses in 21 unique comparisons. To demonstrate the utility of these data, we identify an sRNA that is upregulated during vaginal colonization and demonstrate that overexpression of this sRNA leads to increased bacterial invasion into host epithelial cells. Finally, to monitor RNA degradation, we perform a transcript stability assay to identify highly stable sRNAs and compare stability profiles of sRNA- and protein-coding genes. Collectively, these data provide a wealth of transcriptomic data for putative sRNAs in GBS and a platform for future mechanistic studies.

## OBSERVATION

Streptococcus agalactiae, or group B Streptococcus (GBS), is a Gram-positive bacterium that colonizes the urogenital and gastrointestinal tract of 20% to 30% of healthy individuals (1, 2). However, as an opportunistic pathogen, GBS also causes diverse diseases, including meningitis, sepsis, skin and soft tissue infections, and pneumonia (3, 4). Studies on GBS gene regulation have focused on protein regulators (such as two-component systems), which sense environment signals and respond by regulating bacterial gene expression (5–7). However, nonprotein regulators, such as small, noncoding RNAs (sRNAs), have been understudied in GBS thus far.

Two previous studies identified putative sRNAs in GBS strain NEM316 (8, 9). Pichon et al. utilized *in silico* analysis to identify 197 novel sRNA candidates (8), and subsequent work by Rosinski-Chupin et al. utilized differential RNA sequencing (dRNA-seq) and strand-specific RNA sequencing (RNA-seq) to identify 120 putative sRNAs (9). These sRNAs were later classified by Wolf et al. into conserved RNA families across 27 GBS genomes (10). However, because only those with high structural similarity to known bacterial sRNAs were analyzed, only 30 GBS sRNAs were classified in this study, potentially excluding many legitimate GBS sRNAs. Here, we utilize these previous studies to update the genomes of the clinically relevant strains NEM316 and COH1 to include sRNA annotations and utilize these updated genomes to generate both sRNA expression and transcript stability data.

## ANNOTATION OF sRNAS ON THE GBS GENOME

**FIG 1:**
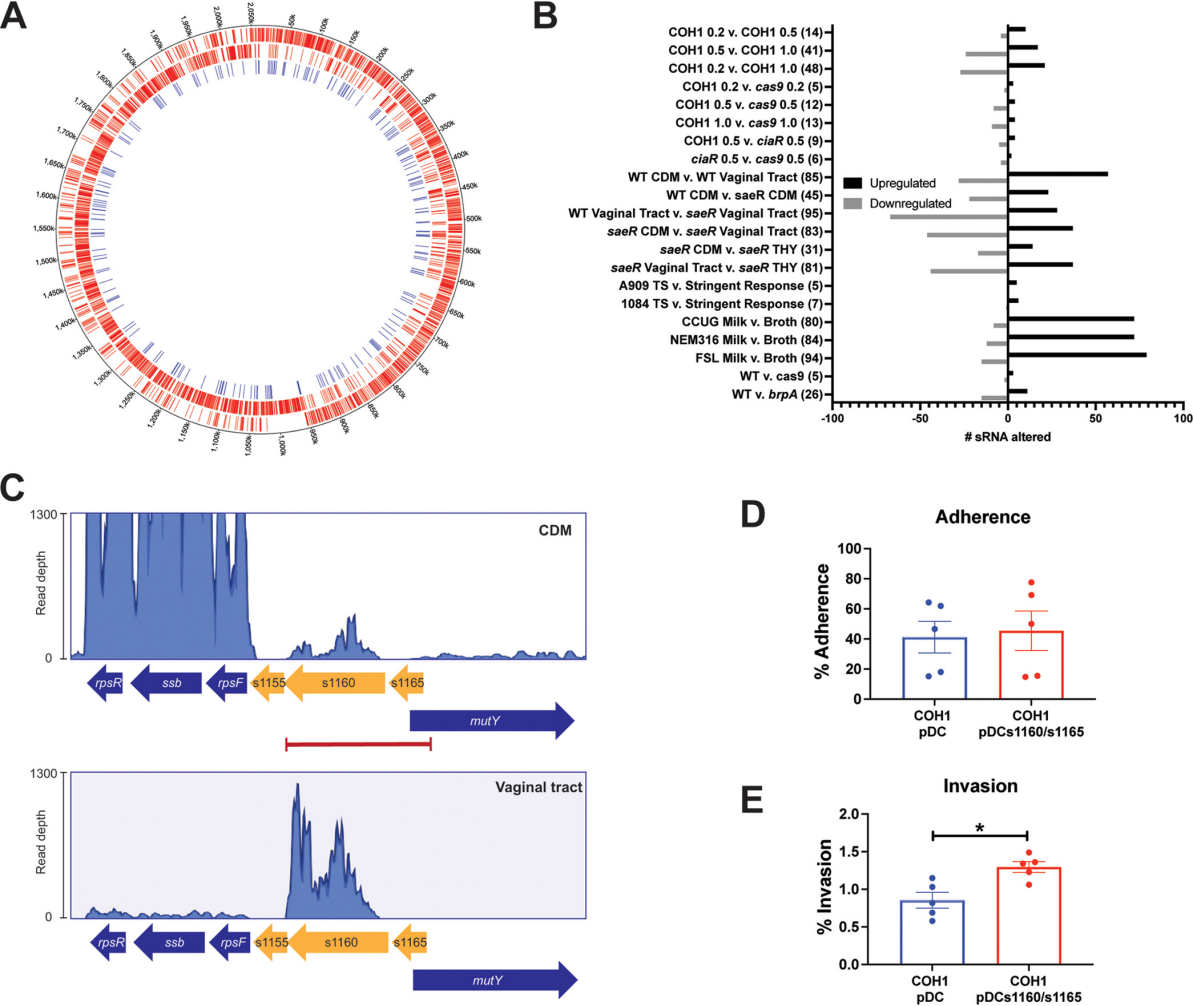
Global sRNA identification and expression analysis. (A) Diagram showing the distribution of sRNA genes (blue lines, inner circle) throughout the GBS genome. Coding sequence genes on the forward strand (outer circle) and reverse strand (middle circle) are depicted in red. Circos plot was created with Circa (http://omgenomics.com/circa). (B) Results of 21 DEAs performed. The conditions for each DEA are indicated as well as the number of sRNA genes upregulated and downregulated (>3-fold). The total number of sRNA genes altered is shown in parentheses. (C). The s1160/s1165 locus and transcript read mapping in GBS strain COH1. Coding sequence genes are illustrated as dark-blue arrows and sRNA genes as yellow arrows. RNA-seq read mappings from DEA9 (CDM top, vaginal tract bottom) are displayed for the locus. Red bar indicates the region cloned into pDC to create pDCs1160/s1165. (D and E) GBS strain COH1 with an empty vector (pDC) compared with an s1160 overexpression strain (pDCs1160/s1165) were used to test adherence to (D) or invasion of (E) hVECs. All data are represented as percent CFU recovered of the initial inoculum and were performed at least in technical triplicates. Each plot is representative of five individual experiments. Significance determined by paired *t* test; *, *P < *0.05.

To facilitate the study of sRNAs in GBS, we updated two genomes from clinically relevant GBS strains (NEM316 and COH1) to include annotations for all putative sRNA candidates identified by Pichon et al. and Rosinski-Chupin et al. Supplemental files indicating the location of the sRNAs from each study were combined, and all repeat-identified sRNAs were eliminated. The remaining sRNAs were then annotated on the GBS genomes, resulting in the addition of 272 sRNAs to the NEM316 genome and 232 to the COH1 genome (Fig. 1A; see File S1 in the supplemental material, genome annotation files available at https://figshare.com/projects/Global_annotation_expression_analysis_and_stability_of_sRNAs_in_Group_B_*Streptococcus*/117768). A total of 40 sRNAs were not added to the COH1 genome due to reduced sequence homology (<80%) or absence from the genome. sRNA-encoding genes were annotated sequentially starting at the origin of replication as was performed for GBS coding sequence annotations (see Text S1).

10.1128/mBio.02803-21.5 FILE S1 Excel file with new sRNA annotations. Download FILE S1, XLSX file, 0.03 MB. Copyright © 2021 Keogh et al. 2021 Keogh et al. https://creativecommons.org/licenses/by/4.0/ This content is distributed under the terms of the Creative Commons Attribution 4.0 International license.

10.1128/mBio.02803-21.10 TEXT S1 Supplemental methods. Download Text S1, DOCX file, 0.03 MB. Copyright © 2021 Keogh et al. 2021 Keogh et al. https://creativecommons.org/licenses/by/4.0/ This content is distributed under the terms of the Creative Commons Attribution 4.0 International license.

## GLOBAL sRNA EXPRESSION ANALYSIS

All previously published GBS RNA-seq studies have overlooked sRNA gene reads. To recover and analyze these overlooked data, we downloaded publicly available GBS RNA-seq data sets and generated expression data for 232 sRNAs in the COH1 background (see Text S1 for selection criteria used). A total of 70 RNA-seq data sets fulfilled the criteria for reanalysis (see File S2 in the supplemental material) (7, 11–15). Using these studies, we performed 21 unique differential gene expression analyses (DEAs) in which sRNA expression was analyzed across two different conditions (see Table S1 in the supplemental material; Fig. 1B). The expression values of all sRNAs in each DEA are included as File S3 in the supplemental material, and the number of differentially expressed (>3-fold with a minimum expression of 10 in at least 1 condition) sRNAs in each comparison is shown in Fig. 1B and File S4 in the supplemental material.

10.1128/mBio.02803-21.6 FILE S2 Studies and data sets used in RNA-seq reanalysis. Download FILE S2, XLSX file, 0.03 MB. Copyright © 2021 Keogh et al. 2021 Keogh et al. https://creativecommons.org/licenses/by/4.0/ This content is distributed under the terms of the Creative Commons Attribution 4.0 International license.

10.1128/mBio.02803-21.4 TABLE S1 Twenty-one DEAs and dataset information. Download Table S1, DOCX file, 0.02 MB. Copyright © 2021 Keogh et al. 2021 Keogh et al. https://creativecommons.org/licenses/by/4.0/ This content is distributed under the terms of the Creative Commons Attribution 4.0 International license.

10.1128/mBio.02803-21.7 FILE S3 Expression values for all sRNA genes in 21 pairwise analyses. Also includes average expression of sRNA and number with a minimum fold change of >3. Download FILE S3, XLSX file, 0.01 MB. Copyright © 2021 Keogh et al. 2021 Keogh et al. https://creativecommons.org/licenses/by/4.0/ This content is distributed under the terms of the Creative Commons Attribution 4.0 International license.

10.1128/mBio.02803-21.8 FILE S4 sRNA genes demonstrating >3-fold variation in expression in 21 pairwise analyses. Download FILE S4, XLSX file, 0.01 MB. Copyright © 2021 Keogh et al. 2021 Keogh et al. https://creativecommons.org/licenses/by/4.0/ This content is distributed under the terms of the Creative Commons Attribution 4.0 International license.

Of these 21 comparisons, we first examined DEA 9 (A909 chemically defined medium [CDM] versus A909 vaginal tract) to evaluate sRNAs that have altered expression *in vivo* (compared with laboratory conditions), as these sRNAs may affect GBS host persistence. A total of 85 sRNAs demonstrated >3-fold variation in expression (which met our cutoff criteria), with 28 being downregulated and 57 being upregulated in the vaginal tract (Fig. 1B; File S4). Of those 57 sRNAs, s1160 was the most highly upregulated in the vagina (88-fold) (Fig. 1C) and exhibited the second highest expression in the GBS cell (average reads per kilobase per million [RPKM] expression level of >32,000 across all conditions examined) (File S3). Since sRNA function is often related directly to abundance, we hypothesized that s1160 may play a role during GBS vaginal colonization. To examine this hypothesis, we overexpressed s1160 in GBS and assessed its interaction (adherence and invasion) with human vaginal epithelial cells (hVECs). The s1160 overexpression construct included the upstream sequence as to include its putative promoter. Although an adjacent sRNA, s1165, was also included in this upstream region, s1165 had an expression value of 0 in the vaginal tract and little to no expression across the other DEAs; therefore, it is unlikely to influence any vaginal colonization phenotypes. s1160 overexpression did not impact GBS adherence to hVECs but did significantly increase GBS invasion compared with the vector control (Fig. 1D and E). These results demonstrate the utility of the analysis presented here and suggest a potential role for s1160 during GBS vaginal colonization. Future studies will examine the contribution of s1160 to GBS colonization *in vivo*, as well as investigate the mechanism of action of this sRNA. Quantitative PCR (qPCR) analysis of the s1160 and s1165 genes in COH1 at an optical density at 600 nm (OD_600_) of 0.2, 0.5, and 1.0 (corresponding to the time points used in DEAs 1, 2, and 3) confirmed the expression patterns observed in the RNA-seq data analysis (see Fig. S1 in the supplemental material).

10.1128/mBio.02803-21.1 FIG S1 Reverse transcriptase quantitative PCR (RT-qPCR) confirmation of s1160 and s1165 growth-phase expression. RT-qPCR analysis of s1160 confirmed increased expression at later timepoints, while s1165 expression was unaltered. Results are similar to those obtained by RNA-seq analysis. Download FIG S1, TIF file, 1.0 MB. Copyright © 2021 Keogh et al. 2021 Keogh et al. https://creativecommons.org/licenses/by/4.0/ This content is distributed under the terms of the Creative Commons Attribution 4.0 International license.

## GLOBAL RNA STABILITY ANALYSIS

**FIG 2:**
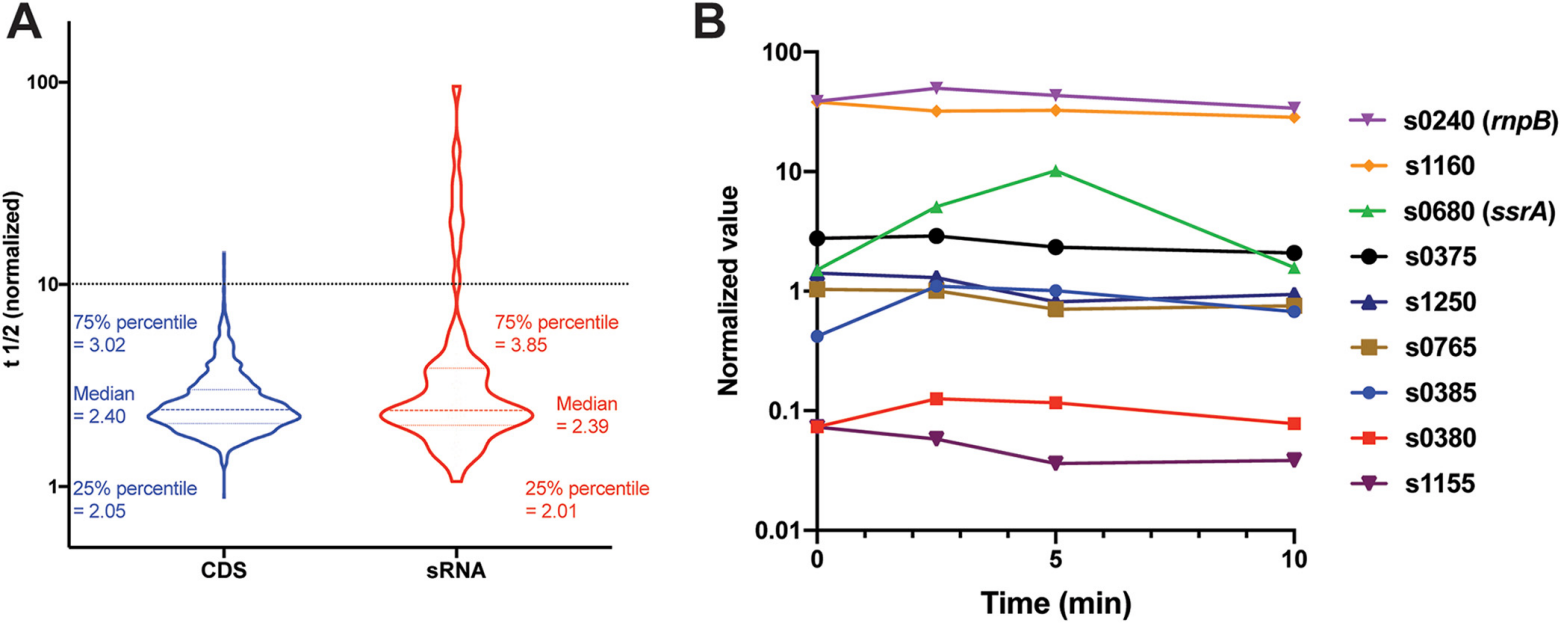
RNA stability analysis. (A) Violin plot of normalized RNA stability data showing (i) the range and (ii) the median half-life of GBS sRNA and coding sequence (CDS) RNA. The 75% and 25% range are also indicated. The dashed line indicates a half-life of 10 min. (B) Normalized stability profiles of select sRNAs over time. Normalized stability plots over time (from 0 to 10 min) are shown for all sRNAs with a half-live exceeding 10 min, including s0385 which had a half-life greater than that of *ssrA*. Plots show abundance relative to *ssrA*.

Cellular RNA levels are regulated by the control of RNA synthesis (i.e., transcription) but also by the rate of RNA stability/degradation. The combined application of rifampicin RNA stability experiments with RNA-seq (here referred to as stability RNA-seq) allows a global analysis of the stability/rate of degradation of all cellular transcripts simultaneously (16). We performed stability RNA-seq using our updated GBS genome reference file and analyzed the stability of both coding DNA sequence (CDS) and sRNA transcripts. Gene expression values were normalized against the value for *ssrA* at the corresponding time point, and the half-life of each RNA was calculated (see Text S1 for details and cut off criteria applied). Half-lives were determined for 1,759 CDS transcripts and 72 sRNAs (see File S5 in the supplemental material). While the median normalized half-life for CDS and sRNA transcripts was very similar (2.4 min and 2.39 min, respectively), the mean half-live for sRNA transcripts was much larger (6.3 min compared with 2.7 min for CDS) (Fig. 2A). Only five CDS transcripts had half-lives exceeding 10 min, with the maximum half-life of 14.4 min for GBSCOH1_RS09545. In contrast, eight sRNA transcripts had half-lives longer than 10 min (Fig. 2B), including values of 45.7 min for *ssrA*, 32.8 min for *rnpB*, and a maximum value of 95.9 min for the uncharacterized sRNA s0380. Of note, s1160 (shown above as one of the most highly expressed sRNAs in GBS and being upregulated during vaginal colonization) was also among the most stable transcripts in the cell with a half-life value of 26.75 min. This result was confirmed by Northern blotting (see Fig. S2 in the supplemental material).

10.1128/mBio.02803-21.9 FILE S5 Normalized rifampicin RNA stability results. Download FILE S5, XLSX file, 0.02 MB. Copyright © 2021 Keogh et al. 2021 Keogh et al. https://creativecommons.org/licenses/by/4.0/ This content is distributed under the terms of the Creative Commons Attribution 4.0 International license.

10.1128/mBio.02803-21.2 FIG S2 Northern blot confirming the stability of s1160 following rifampicin treatment. RNA was isolated from GBS cultures treated with rifampicin for the indicated time. Blots were probed with labeled oligonucleotides antisense to s1160 (top) and RS03030 as a positive control (bottom). Detection of s1160 was possible at all timepoints up to and including 30 min after rifampicin treatment. In contrast, a band corresponding to RS03030 (which was shown to be highly unstable by RNA-seq) was detected only at 0 mins (i.e., prior to rifampicin addition). Download FIG S2, TIF file, 1.9 MB. Copyright © 2021 Keogh et al. 2021 Keogh et al. https://creativecommons.org/licenses/by/4.0/ This content is distributed under the terms of the Creative Commons Attribution 4.0 International license.

The half-life analysis returned negative values for three CDS transcripts and five sRNAs. In most cases, this result was because one (or more) of the normalized expression values for that transcript was zero at one (or more) time points. However, one CDS and one sRNA transcript, namely, GBSCOH1_RS10860 and GBSCOH1_s0385, generated negative values because their normalized expression values increased over time, indicating that the abundance of these transcripts increased relative to *ssrA*; therefore, they are more stable than *ssrA*. Interestingly, s0385 (which exhibited the highest sRNA half-life) (Fig. 2B) is encoded adjacent but antisense to another highly stable sRNA, s0380. These two sRNAs are divergently transcribed, and the locus contains a gene potentially encoding a holin-like protein (see Fig. S3 in the supplemental material). The high degree of stability of antisense transcripts, of which one putatively encodes a toxin, is reminiscent of a toxin-antitoxin system and would be of interest to study in the future.

10.1128/mBio.02803-21.3 FIG S3 The s0380/s0385 genomic locus in GBS. Orientation and positions of the s0380 and s0385 genes are indicated by blue arrows. Location of an open reading frame potentially encoding a holin homologue is indicated by the yellow arrow. Download FIG S3, TIF file, 0.2 MB. Copyright © 2021 Keogh et al. 2021 Keogh et al. https://creativecommons.org/licenses/by/4.0/ This content is distributed under the terms of the Creative Commons Attribution 4.0 International license.

## CONCLUSIONS

The data presented here demonstrate the utility of the updated genome annotation files, allowing us to determine the stability of 232 sRNAs in COH1 as well as their expression in 21 unique comparisons. These results will inform future phenotypic studies and likely identify new sRNA regulators in GBS. Undoubtedly, many sRNAs have yet to be discovered in GBS. Our newly annotated genomes will be an invaluable tool for the further identification of sRNAs, and these updated annotation files will prevent repeat sRNA identification in future studies. Collectively, the data generated highlight the importance of updating genome annotation files as new regulatory elements are identified in bacteria.
