# Supplementary material for: Global Annotation, Expression Analysis, and Stability of Candidate sRNAs in Group B Streptococcus
Source: mBio. 2021 Nov 2;12(6):e02803-21. doi: 10.1128/mBio.02803-21 (PMC8561379; doi:10.1128/mBio.02803-21)
Supplement: TEXT S1 [file mbio.02803-21-s0006.docx]

**SUPPLEMENTAL MATERIALS AND METHODS**

**sRNA nomenclature**

In GBS strain COH1, nomenclature for sRNA annotations utilized the prefix “GBSCOH1_s” followed by a numerical value (e.g. GBSCOH1_s0005), while in NEM316 the prefix “GBSNEM316_s” was used. Numerical values for sRNA genes increased sequentially starting at the origin of replication in intervals of 5 based upon the established GBS annotation system for coding DNA sequence (CDS) genes. This system accounts for the possibility of future sRNA identification without disrupting the continuous numbering.

**RNA-seq data acquisition**

We first searched “*Streptococcus agalactiae*” on the Sequence Read Archive on NCBI and employed the following five criteria to determine which studies and datasets would be included in this re-analysis: (i) samples were from the GBS species, (ii) samples were provided in at least two replicates, (iii) samples were complete transcriptomic data sets (and not dRNA-seq or another variant), (iv) samples could be placed into comparable groups (i.e. parental strain vs. mutant, or different phases of growth), and (v) no previous analysis of GBS sRNAs had been performed with the same datasets. Six studies, encompassing a total of 70 RNAseq datasets, fulfilled the criteria for re-analysis (Table 2, Supplementary file 5) (1–6).

**Bacterial strains and cell lines**

*Escherichia coli* strain MC1061 (Mclab) was used to propagate the pDCErm empty vector plasmid and the pDCs1160 overexpression plasmid, pDCErm-containing *E. coli* strains were grown in LB (Lysogeny broth; Research Products International, RPI) supplemented with 500 µg/ml erythromycin at 37 °C, shaking. All GBS strains were made in the ST-17/serotype III clinical isolate background of COH1 (7) and were grown in Todd Hewitt Broth (THB; Research Products International, RPI), statically at 37 °C (with 5 µg/ml erythromycin). Immortalized hVEC human vaginal epithelial cells (hVEC) were obtained from the American Type Culture Collection (VK2.E6E7, ATCC CRL-2616) and were maintained in keratinocyte serum-free medium (KSFM; Gibco) with 0.1 ng/ml human recombinant epidermal growth factor (EGF; Gibco) and 0.05 mg/ml bovine pituitary extract (Gibco) at 37 °C with 5% CO_2._

**Construction of GBS vector control and s1160 overexpressing strains**

Construction of GBS vector control and complemented strains was performed as described previously (1). The s1160 overexpressing-COH1 strain was generated by PCR amplification of s1160 and its upstream ~200 base-pairs (to include the putative native promoter) using primers 5084 (atatatccgcggTACCGTATTAACTTGTGTTTGTTGC) and 3080 (atatatggatccAATAAAAAGGCGAGGTTTACACC). The s1160 construct and overexpression vector pDCErm were digested with SacII and BamHI (NEB), ligated via Quick Ligase (NEB), and transformed into MC1061 competent cells (McLab) at 37 °C. Positive transformants were selected by growth on LB+ 500 μg/ml erythromycin at 37 °C and were PCR confirmed using 5012 (TGGGTCAATCGAGAATATCGTC) and pDCErmrev2 (CTTGTTGCCCAACTAAATAGG) primers. pDCs1160/s1165 and the pDC empty vector were then maxi-prepped (Qiagen) and transformed into COH1 competent cells. Positive transformants were selected by resistance to 5 μg/ml erythromycin and confirmed by PCR and DNA sequencing.

**Adherence and invasion of GBS to host cells.**

Adherence and invasion assays were performed as previously described (1). hVEC monolayers were infected at a Multiplicity of infection (MOI) of 1 with GBS grown to mid-log phase and normalized in PBS. To assess adherence of GBS to host cells, bacteria were incubated with hVEC monolayers for 30 min. To assess bacterial invasion, GBS was incubated with hVEC for 2 h, the monolayer was washed with PBS and then incubated with medium containing antibiotics for 2 h to kill extracellular bacteria. At the end of each assay, media was removed and the cell monolayers were washed with PBS, detached with 0.25 % trypsin (Thermo Fisher Scientific), and permeabilized with 0.025 % Triton X-100 (Sigma) in PBS. Cell lysates were serially diluted and plated to quantify all hVEC-associated or hVEC-invaded bacteria, respectively.

**Stability RNA-seq**

GBS was grown to mid-exponential phase and an aliquot of cells withdrawn for RNA isolation (T=0 timepoint). Rifampicin (which halts de novo transcription) was added to a final concentration of 200 μg/ml, and further aliquots of bacterial cells taken 2.5-, 5-, and 10-min post rifampicin-treatment (T= 2.5, 5, and 10). Following RNA extraction, RNA-seq was performed. Raw RNA-seq expression values were generated for each gene and lowly expressed genes (RPKM value < 10) were eliminated. In stability RNA-seq data analysis, to account for the overall reduction in RNA over time, raw gene expression values are typically normalized against a highly expressed, highly stable transcript, such as *hup* (which encodes the nucleoid associated protein HU). Our RNA-seq data indicated that *hup* is less highly expressed and less stable in GBS than in other Gram-positive bacteria; therefore, we instead normalized data against the highly expressed and highly stable *ssrA* transcript which encodes tmRNA.

**RNA decay constant and half-live determination**

Calculation of RNA half-life was made as previously described (8). Transcripts with a normalized expression value less than 10 at T0 and < 80% of reads mapping to unique genes were eliminated. Each transcript was normalized against the endogenous control ssrA at each time points. RNA half-life was calculated according to the following equation: $t_{1/2}=ln2/k_{decay}$ with $k_{decay}$ the rate constant for decay, obtained from the slope of a semi-logarithmic plot of normalized values of each transcript as a function of time. All analysis was performed with RStudio v1.4 (9).

**Northern blotting**

Northern blotting was performed as described previously (10). Bacterial cultures were treated with rifampicin, as outlined above, and RNA isolated 0-, 2.5-, 5-, 10-, 20- and 30-min post rifampicin treatment. 3 μg of RNA was loaded in each lane of a 1.2% agarose gel containing formaldehyde. Following electrophoresis RNA was transferred to nylon membrane and pre-hybridized with DIG easy hyb solution. Blots were probed with single stranded biotinylated oligos antisense to s1160 (CCT ACA TCT AAA CCT AAC ATA AGT GCC CCT C) or RS03030 (GAC GTC CTG TAT AGA ATG GGT GTG AAT CTG) overnight at 42 °C. Following the overnight hybridization blots were washed once with 2X SSC, once with 1X SSC and once with 0.5X SSC (each wash 10 min at 42 °C). Blots were subsequently detected using the Chemiluminescent Nucleic acid Detection kit (Pierce) according to the manufacturer’s instructions and visualized using a Chemidoc CCD camera (Biorad).

**qRT-PCR Confirmation of RNA-Sequencing**

qRT-PCR was performed as previously described (11) to confirm the expression of s1160 and s1165 observed in the temporal RNA-Seq analysis. Bacterial strains were grown in triplicate to mid-log phase and bacterial RNA was isolated following the manufacturer’s protocol using the Direct-Zol RNA MiniPrep Plus kit (Zymo Research). cDNA was generated using the Quanta cDNA synthesis kit (Quanta biosciences) and transcript abundance was determined using PerfeCTa SYBR Green reagent. Fold changes in transcript abundance were calculated using ∆∆CT, by which target gene transcript levels were normalized to those of housekeeping gene, *gyrA*. qRT-PCR data represent the average of three biological replicates qRT-primers used in this study are listed below.

s1160 F: AATATGGGTCGCTATAAGAATGGC

s1160 R: GGACTTATGTGTCACCGTTACTG

s1165 F: TAGTAGTTCTTCGCCAAGGGAG

s1165 R: CAACATACCTCTTTGTCAAATCCTC

*gyrA* F: AGCACAAAAACGTGGAGGAC

*gyrA* R: ACGATAGGGAGGCCTTTAGC

References

1. Spencer BL, Deng L, Patras KA, Burcham ZM, Sanches GF, Nagao PE, Doran KS. 2019. Cas9 contributes to group b streptococcal colonization and disease. Front Microbiol 10:1–15.

2. Cook LCC, Hu H, Maienschein-cline M, Federle J. 2018. A Vaginal Tract Signal Detected by the Group B Streptococcus SaeRS System Elicits Transcriptomic Changes and Enhances. Infect Immun 1–17.

3. Hooven TA, Catomeris AJ, Bonakdar M, Tallon LJ, Santana-cruz I, Ott S, Daugherty SC, Tettelin H, Ratner J. 2018. The Streptococcus agalactiae Stringent Response Enhances Virulence and Persistence in Human Blood. Infect Immun 1–15.

4. Richards VP, Choi SC, Bitar PDP, Gurjar AA, Stanhope MJ. 2013. Transcriptomic and genomic evidence for Streptococcus agalactiae adaptation to the bovine environment. BMC Genomics 14:1–15.

5. Ma K, Cao Q, Luo S, Wang Z, Liu G, Lu C, Liu Y. 2018. cas9 enhances bacterial virulence by repressing the regR transcriptional regulator in Streptococcus agalactiae. Infect Immun 86.

6. Patras KA, Derieux J, Al-Bassam MM, Adiletta N, Vrbanac A, Lapek JD, Zengler K, Gonzalez DJ, Nizet V. 2018. Group B Streptococcus Biofilm Regulatory Protein A Contributes to Bacterial Physiology and Innate Immune Resistance. J Infect Dis 218:1641–1652.

7. Kuypers JM, Heggen LM, Rubens CE. 1989. Molecular analysis of a region of the group B streptococcus chromosome involved in type III capsule expression. Infect Immun https://doi.org/10.1128/iai.57.10.3058-3065.1989.

8. Chen CA, Ezzeddine N, Shyu A. Messenger RNA Half-Life Measurements in Mammalian CellsRNA Turnover in Eukaryotes: Nucleases, Pathways and Anaylsis of mRNA Decay, 1st ed. Elsevier Inc.

9. R Core Team (2018) R: A Language and Environment for Statistical Computing. R Foundation for Statistical Computing, Vienna.

10. Zapf RL, Wiemels RE, Keogh RA, Holzschu DL, Howell KM, Trzeciak E, Caillet AR, King KA, Selhorst SA, Naldrett MJ, Bose JL, Carroll RK. 2019. The small RNA Teg41 regulates expression of the alpha phenol-soluble modulins and is required for virulence in staphylococcus aureus. MBio 10.

11. Liwen Deng, Rong Mu, Thomas A. Weston, Brady L. Spencer, Roxanne P. Liles Kelly, S. Doran. 2018. Characterization of a Two-Component System Transcriptional Regulator, LtdR, That Impacts Group B Streptococcal Colonization and Disease 86:1–17.
