# Supplementary material for: Global Annotation, Expression Analysis, and Stability of Candidate sRNAs in Group B Streptococcus
Source: mBio. 2021 Nov 2;12(6):e02803-21. doi: 10.1128/mBio.02803-21 (PMC8561379; doi:10.1128/mBio.02803-21)
Supplement: TABLE S1 [file mbio.02803-21-st001.docx]

Table S1. Pairwise comparisons conducted in this study.

| Study | Pairwise comparison | Exp no. | Details | No. of sRNAs altered (min 3-fold) |
| --- | --- | --- | --- | --- |
| 1 | 1 | 1.1 | COH1 0.2 v. COH1 0.5 | 14 |
| 1 | 2 | 1.2 | COH1 0.5 v. COH1 1.0 | 41 |
| 1 | 3 | 1.3 | COH1 0.2 v. COH1 1.0 | 48 |
| 1 | 4 | 1.4 | COH1 0.2 v. *cas9* 0.2 | 5 |
| 1 | 5 | 1.5 | COH1 0.5 v. *cas9* 0.5 | 12 |
| 1 | 6 | 1.6 | COH1 1.0 v. *cas9* 1.0 | 13 |
| 1 | 7 | 1.7 | COH1 0.5 v. *ciaR* 0.5 | 9 |
| 1 | 8 | 1.8 | *ciaR* 0.5 v. *cas9* 0.5 | 6 |
| 2 | 9 | 2.1 | WT CDM v. WT vaginal tract | 85 |
| 2 | 10 | 2.2 | WT CDM v. *saeR* CDM | 45 |
| 2 | 11 | 2.3 | WT vaginal Tract v. *saeR* vaginal tract | 95 |
| 2 | 12 | 2.4 | *saeR* CDM v. *saeR* vaginal tract | 83 |
| 2 | 13 | 2.5 | *saeR* CDM v. *saeR* THY | 31 |
| 2 | 14 | 2.6 | *saeR* vaginal tract v. *saeR* THY | 81 |
| 3 | 15 | 3.1 | A909 TS v. Stringent Response | 5 |
| 3 | 16 | 3.2 | 1084 TS v. Stringent Response | 7 |
| 4 | 17 | 4.1 | CCUG Milk v. Broth | 80 |
| 4 | 18 | 4.2 | NEM316 Milk v. Broth | 84 |
| 4 | 19 | 4.3 | FSL Milk v. Broth | 94 |
| 5 | 20 | 5.1 | WT v. *cas9* mutant | 5 |
| 6 | 21 | 6.1 | WT v. *brpA* mutant | 26 |
