# Supplementary figures and images for: Global Annotation, Expression Analysis, and Stability of Candidate sRNAs in Group B Streptococcus
Source: mBio. 2021 Nov 2;12(6):e02803-21. doi: 10.1128/mBio.02803-21 (PMC8561379; doi:10.1128/mBio.02803-21)

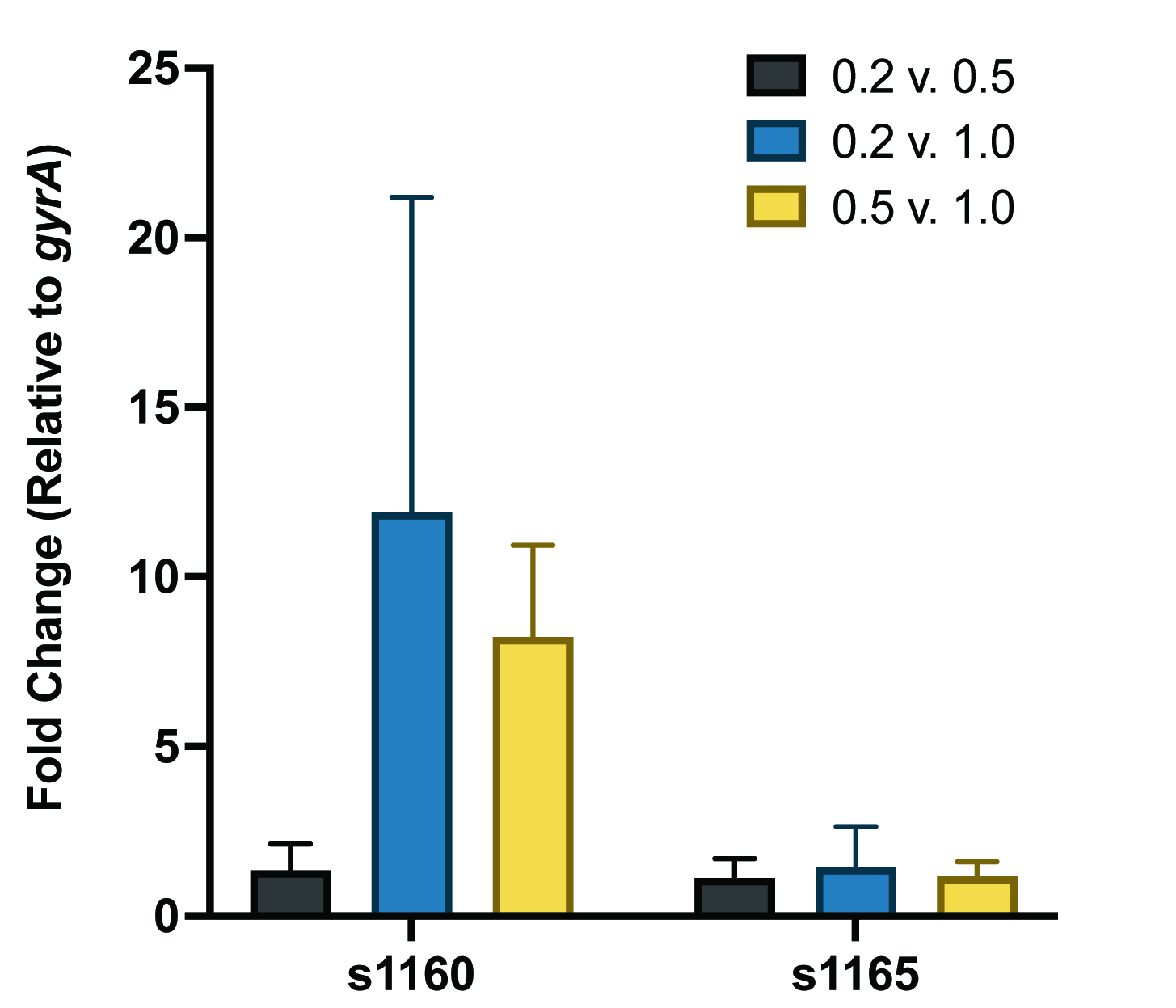

Supplement: FIG S1 [file mbio.02803-21-sf001.tif]

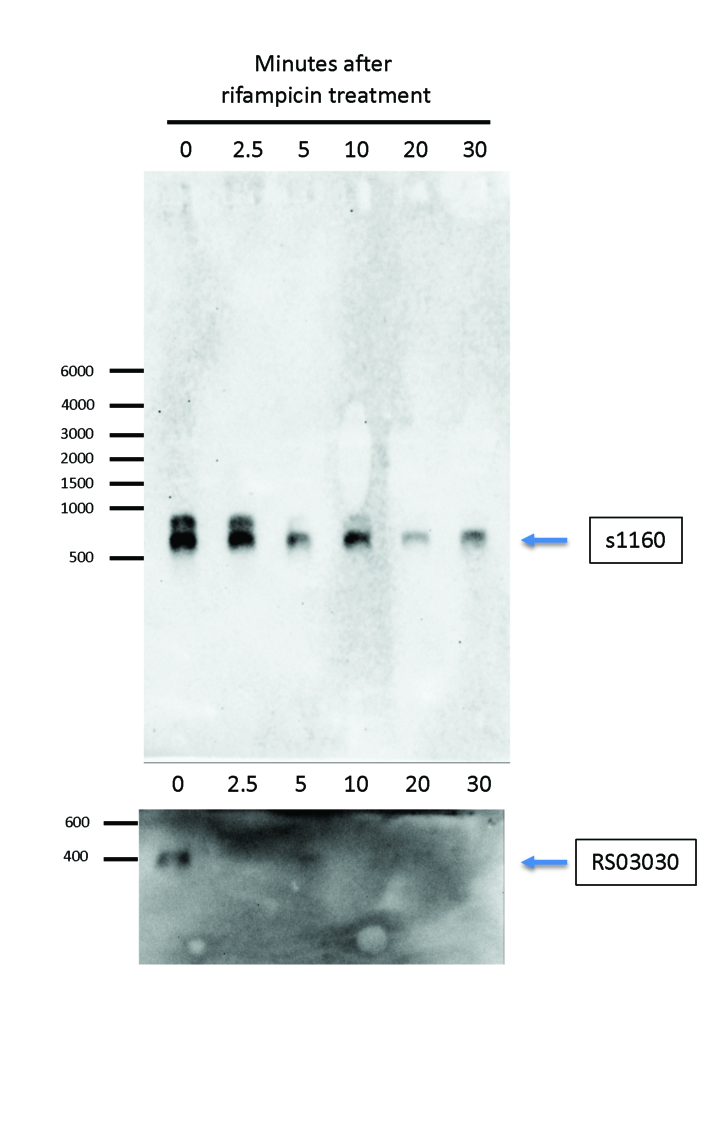

Supplement: FIG S2 [file mbio.02803-21-sf002.tif]

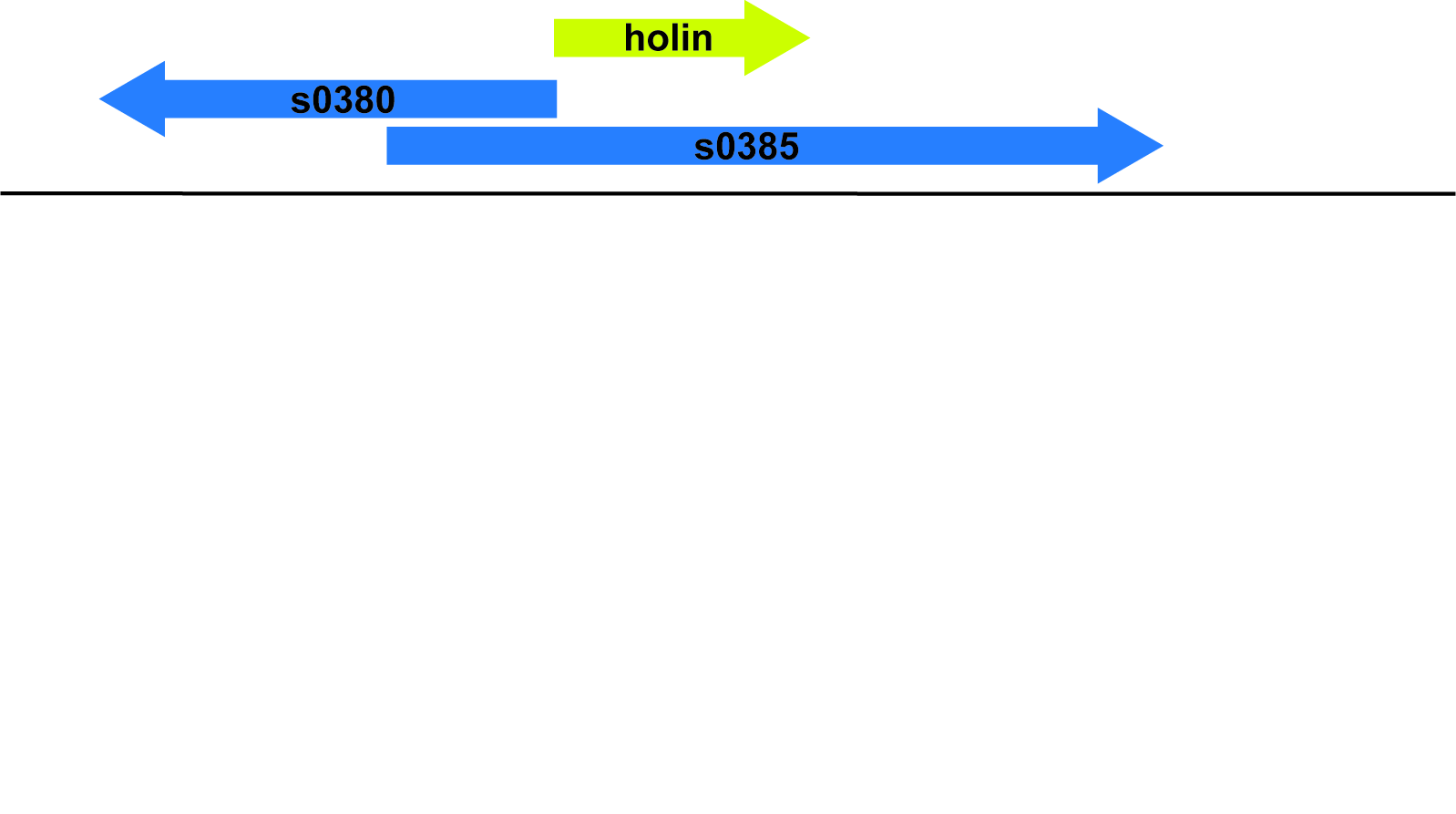

Supplement: FIG S3 [file mbio.02803-21-sf003.tif]
